# Maduración esquelética de mano y muñeca y osificación de sutura media palatina en adolescentes: una revisión de literatura

**DOI:** 10.21142/2523-2754-1103-2023-167

**Published:** 2023-09-26

**Authors:** Ruth Elizabeth Ramirez-Diaz, Gustavo Adolfo Watanabe Kanno

**Affiliations:** 1 . Division of Orthodontic, School of Dentistry, Universidad Científica del Sur. Lima, Perú. 100069814@cientifica.edu.pe, adolfwk@yahoo.com Universidad Científica del Sur Division of Orthodontic School of Dentistry Universidad Científica del Sur Lima Peru 100069814@cientifica.edu.pe adolfwk@yahoo.com

**Keywords:** maduración esquelética, análisis de mano y muñeca, sutura media palatina, skeletal maturation, hand and wrist analysis, midpalatal suture

## Abstract

**Introducción:**

La evaluación de la edad esquelética es un factor importante en la planificación ortodóncica para anticipar cambios en el crecimiento y es el análisis de radiografías de mano y muñeca el que muestra el grado del potencial de crecimiento óseo y facial. El objetivo fue evaluar la relación entre la maduración esquelética de la mano y muñeca, y la osificación de sutura media palatina (SMP) en adolescentes.

**Materiales y métodos:**

Se realizó una búsqueda en cuatro bases de datos: PubMed, Scopus, ScienceDirect y Embase, revisadas hasta el 13 de diciembre de 2022. Los estudios incluidos fueron artículos descriptivos y comparativos de la maduración esquelética de la mano y muñeca, y osificación de la sutura media palatina de pacientes de 7 a 18 años. Dos investigadores seleccionaron cuidadosamente los artículos evaluados y analizaron los diferentes tópicos clave relacionados con el tema.

**Resultados:**

En este estudio se incluyeron 4 artículos. Según los estudios, se encontró que mientras mayor sea el grado de maduración ósea hay un aumento en la aproximación de la SMP, especialmente en etapas tardías, con correlaciones altas y positivas; además, hubo mayores resultados de correlación con el método de análisis de Fishman, a diferencia de los métodos de Hagg y Taranger y Björk. En los estadios de límite critico en SMI7-9, se encontró mayor acercamiento al cierre de SMP compatibles con estadio D-E. La finalización de la maduración en mujeres se completó hasta en 2 años antes que en los hombres.

**Conclusiones:**

Los métodos de evaluación para el diagnóstico mediante el análisis carpal puede usarse para las evaluaciones de predicción del estadio de maduración de la SMP; sin embargo, los resultados no fueron absolutos en todos los casos, por lo que no se puede generalizar.

## Abstract (en)

**Introduction:**

The evaluation of skeletal age is an important factor in orthodontic planning to anticipate changes in growth, with the analysis of hand and wrist radiographs showing the degree of bone and facial growth potential. The objective was to evaluate the relationship between skeletal maturation of the hand and wrist and ossification of the midpalatal suture (MPS) in adolescents**.**

**Materials and methods:**

A search was carried out in four databases such as Pubmed, Scopus, Science Direct and Embase were reviewed until December 13, 2022. The included studies were descriptive and comparative articles on the skeletal maturation of the hand and wrist and ossification of the midpalatal suture of patients aged 7 to 18 years. Two researchers carefully selected the articles evaluated and analyzed the different key topics related to the topic.

**Results:**

Four articles were included in this study; According to the studies, it was found that the greater the degree of bone maturation there is an increase in the approximation of the SMP, especially in late stages, with high and positive correlations; Furthermore, there were greater evaluation results with the Fishman analysis method as opposed to the Hagg and Taranger and Björk methods. The critical limit stages in SMI7-9, a greater approach to the closure of SMP compatible with stage D-E was found. The completion of maturation in women occurs up to 2 years earlier than in men.

**Conclusions:**

Diagnostic evaluation methods using carpal analysis can be used for predictive evaluations of the maturation stage of SMP; However, the results were not absolute in all cases so they cannot be generalized.

## INTRODUCCIÓN

El ser humano pasa por distintos períodos durante su crecimiento y desarrollo, lo que involucra cambios característicos e individuales que son influenciados por factores genéticos y ambientales. Su evaluación se puede realizar mediante varios indicadores, como edad cronológica, desarrollo dental, características sexuales secundarias, velocidad máxima de crecimiento y maduración esquelética ^1^^-^^4^. La evaluación de la edad esquelética es un factor importante en la planificación de la terapia para anticipar cambios en el crecimiento, y es lógico esperar que los niveles de maduración esquelética varíen con los diferentes patrones esqueléticos ^5^^-^^8^. 

En cuanto a las técnicas de diagnóstico más utilizadas, tenemos la radiografía de mano y muñeca, descrita por Fishman, y los estadios de osificación de las vértebras cervicales, descritos por Baccetti ^1^^,^^2^^,^^9^^,^^10^. El estudio de radiografías de mano y muñeca muestra el grado del potencial de crecimiento óseo y facial, que lleva a cambios de manera secuencial a 4 etapas de osificación: cambio del ancho en la epífisis de la falange, osificación del sesamoideo, blindaje de la epífisis hacia la diáfisis y anexión de la epífisis ^11^^,^^13^. Por lo tanto, demuestra ser un enfoque ampliamente utilizado para predecir el momento del crecimiento máximo puberal y la estimación de la velocidad de crecimiento y la proporción de crecimiento residual ^14^^-^^16^.

La sutura media palatina (SMP) se considera un tipo de sutura de extremo a extremo, con características específicas de su morfología que se ven durante el crecimiento. El comienzo y el progreso de la fusión de la sutura varían mucho con la edad y el sexo. Sin embargo, lo que se pretende conseguir es la separación de la sutura palatina y las suturas circundantes del maxilar, que se realizan para la corrección de las maloclusiones como las mordidas cruzadas posteriores y el apiñamiento. La terapia se ha limitado a pacientes en crecimiento, ya que el fracaso clínico se da especialmente en adultos jóvenes, que llegan a presentar complicaciones ^12^^,^^17^^-^^19^. 

La importancia de este estudio radica en evidenciar la etapa del crecimiento solo con observar los cambios radiográficos de la mano y muñeca, a fin de pronosticar el grado de osificación de la sutura media palatina y viceversa, además de disminuir la exposición a la radiación, tiempo y costo. Por ello, el propósito fue relacionar la maduración esquelética de la mano y muñeca con la sutura media palatina, mediante el uso de radiografía carpal y CBCT de la sutura media palatina.

## MATERIALES Y MÉTODOS

### Estrategia de búsqueda

La búsqueda del material bibliográfico fue ejecutada hasta el 13 de diciembre de 2022, en cuatro bases de datos; PubMed, Embase, Scopus y ScienceDirect. El método de exploración se examinó en descriptores MeSH (Medical Subject Heading) y los términos de búsqueda fueron los siguientes: (maturation OR "skeletal maturation" OR "skeletal growth" OR "skeletal age") AND ("carpal analysis" OR hand OR "wrist analysis") AND (midpalatal OR "bone maturation" OR "midpalatal suture"). Las referencias fueron ordenadas mediante la búsqueda de citas Refworks para evitar duplicados (Tabla 1).

**Tabla 1:** Estrategia de búsqueda de descriptores de las diferentes bases de datos

PubMed (13/12/2022)
n = 94
(maturation OR "skeletal maturation" OR "skeletal growth" OR "skeletal age") AND ("carpal analysis" OR hand OR "wrist analysis") AND (midpalatal OR "bone maturation" OR "midpalatal suture")
Scopus (13/12/2022)
n = 139
(maturation OR "skeletal maturation" OR "skeletal growth" OR "skeletal age") AND ("carpal analysis" OR hand OR "wrist analysis") AND (midpalatal OR "bone maturation" OR "midpalatal suture")
Embase (13/12/2022)
n = 116
('maturation':ti,ab,kw OR 'skeletal maturation':ti,ab,kw OR 'skeletal growth':ti,ab,kw OR 'skeletal age':ti,ab,kw) AND ('carpal analysis':ti,ab,kw OR 'hand':ti,ab,kw OR 'wrist analysis':ti,ab,kw) AND ('bone maturation':ti,ab,kw OR 'midpalatal':ti,ab,kw)
ScienceDirect (13/12/2022)
n = 16
(maturation OR "skeletal growth" OR "skeletal age") AND ("carpal analysis" OR hand OR "wrist analysis") AND (midpalatal OR "bone maturation" OR "midpalatal suture")

### Criterios de selección

La estrategia de búsqueda se basó en la pregunta PTCO: P (población de pacientes de 7 a 18 años), T (técnica índice a la osificación de la sutura media palatina), C (comparación con la maduración de la mano y muñeca) y O (resultados del estadio de maduración ósea). Los artículos incluidos fueron ensayos clínicos descriptivos y comparativos de la maduración esquelética de la mano y muñeca, y osificación de la sutura media palatina, que apliquen en estudios radiográficos en individuos de 7 a 18 años, y emitidos en idioma inglés. Se excluyeron los estudios de casos que analicen pacientes con tratamiento de ortodoncia, pacientes con síndrome craneofacial y enfermedades óseas. Tampoco se consideraron estudios como revisiones de literatura, revisiones sistemáticas, reporte de caso(s), estudios sin texto completo, estudios *in vitro*, en cadáveres y en animales.

### Extracción de datos

**Figura 1:**
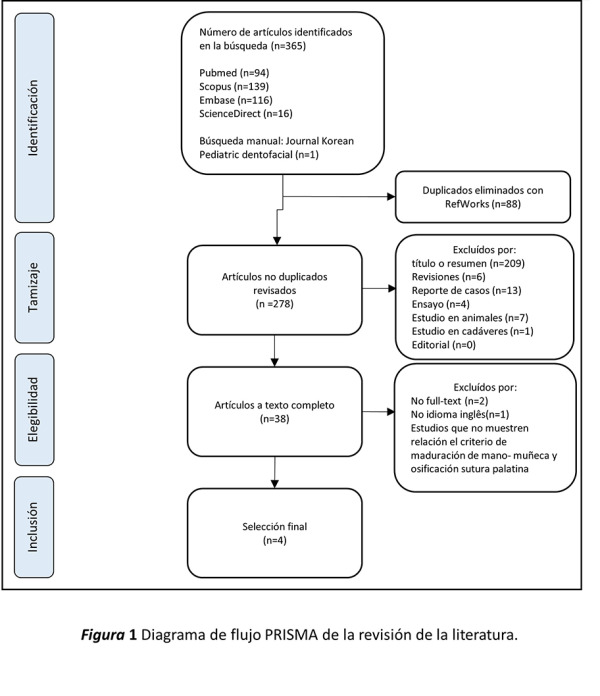
Diagrama de flujo PRISMA de la revisión de la literatura.

Dos investigadores (RERD y GAWK) se calibraron para la selección de los estudios, de acuerdo con los criterios de selección (revisión de 10 artículos). La investigadora (RERD) evaluó los títulos y resúmenes de los estudios utilizando criterios PRISMA (Preferred Reporting Items for Systematic Reviews and Meta-Analyses) (Figura 1). En caso de dudas sobre la inclusión del artículo, se revisó a texto completo y las discrepancias fueron resueltas por el segundo autor (GAWK). 

### Resultados

Los datos reunidos de los artículos fueron el autor, país, año de publicación, criterio de exclusión, muestra (sexo y edad), tipo de evaluación de sutura media palatina, método de evaluación de maduración carpal y resultados. Las características de los artículos seleccionados se colocaron en la tabla de extracción (Tabla 2). La calidad metodológica de estos estudios fue analizada con PRISMA *checklist*.

**Tabla 2:** Tabla de extracción de información

ID	AUTOR, PAÍS, AÑO	DISEÑO DE ESTUDIO	EXCLUSIÓN	TAMAÑO DE MUESTRA	SEXO(n)	RANGO DE EDAD	EVALUACIÓN DE SMP	MÉTODO DE MADURACIÓN CARPAL	RESULTADOS	CONCLUSIONES
1	Akbulut *et al*., Turquía, 2020	Análisis retrospectivo	Pacientes con enfermedades que afectara desarrollo óseo	90	45 v	8,73-18,69 a	Evaluación clínica G1:S-RPE G2: F-RPE	M.B. Radio, DP3, PP3, MP3	Sin considerar sexo ni edad, los valores fractales comparados G1: Radio: 1,179, PP3: 1,180; MP3: 1,138; DP3: 1,086	Valores significativamente diferentes entre G1 y G2.	Los valores fractales comparados conG1-G2 del radio (p < 0,001), MP3 (p < 0,016), DP3 (p < 0,004), PP3 (p < 0,063)	Los valores fractales más bajos en G1 que en G2, con excepción de PP3.	45 m	Los valores fractales aumentaron en G2. Los valores más significativos ocurrieron en el grupo de medición de Radio.
2	Jang *et al*., Corea del Norte, 2016	Análisis retrospectivo	Pacientes con enfermedades o ingesta de medicamentos que afectara desarrollo óseo	99	40 v	v:14,3 a ±3,27;	Imágenes CBCT C.A. (A, B, C, D, E)	A.F. SMI1; SMI2, SMI3; SMI4; SMI5, SMI6; SMI7; SMI8; SMI9; SMI10; SMI11	Correlación intraclase ICC: C. A.: 0,995 (p < 0,05). A. F.: 0,096 (p < 0,05)	Correlación fuerte y significativa entre C. A. y A. F, comparada con otros estudios.	Tratamiento de ortodoncia previo	59 m	m:13,56 ±3,12	Correlación rho de Spearman C. A. y A. F. (0,904). Tabulación cruzada C. A. y A. F. (p < 0,0001), A. F. γ = 0,924 > 0,905, τ-b de Kendall = 0,087 > 0,784.	El análisis de tabulación cruzada según el sexo y A. F. mostró valores significativamente altos comparado con otros estudios.
3	Revelo *et al*., EE. UU., 1994	Análisis retrospectivo	Pacientes con enfermedades que afectara desarrollo óseo	84	39 v	8-18 a	Radiografías oclusales A-P, A-B, B-P	A.F. SMI1; SMI2, SMI3; SMI4; SMI5, SMI6; SMI7; SMI8; SMI9; SMI10; SMI11	SMI1 (1,1; rg:4,4); SMI2 (2,7; rg:0,0-7,0), SMI3 (8,0; rg 6,2-10,5); SMI4 (13,0; rg 11,2-20,0); SMI5 (16,0; rg11,7-20,5), SMI6 (16,8; rg12,5-27,5); SMI7 (22,9; rg:13,4-30,0); SMI8 (24,4; rg 22,8-24,5); SMI9 (26,5; rg:21,2-35,2); SMI10 (45,1; rg:28,0-62,3); SMI11 (30,0; rg:30,0-69,0).	Mientras aumenta la maduración ósea, se observa aumento en la aproximación de la sutura media palatina, especialmente en las etapas tardías de maduración. SMI9 aumento significativo.	45 m	Correlación: m: AP(0.869), BP(0.930), AB(0.665); v: AP(0.920), BP(0.926), AB(0.713). p < 0,0001	Valores altamente correlacionados en la parte posterior de la sutura (B-P) con la etapa de maduración ósea. Mayor variabilidad en la parte anterior de la sutura(A-B).
4	Yu *et al*., Corea del Norte, 2021	Análisis retrospectivo	Pacientes con enfermedades o ingesta de medicamentos que afectara desarrollo óseo	267	132v	7-15 a	Imágenes CBCT C.A. (A, B, C, D, E)	A.F. SMI1; SMI2, SMI3; SMI4; SMI5, SMI6; SMI7; SMI8; SMI9; SMI10; SMI11	C. A. y A. F.: coeficiente de correlación de Spearman (ϒs = 0,905, p < 0,05)	Correlaciones positivas y estadísticamente significativas, ligeramente mayor para A. F.	Tratamiento de ortodoncia previo	135m	H. T MP3 (MP3F, MP3G, MP3G, MP3H, MP3I)	C. A. y H. T.: (ϒs = 0830, p < 0,05)	Correlaciones positivas y estadísticamente significativas.

m: mujeres; v: varones; a: años; S-RPE: apertura sutura media palatina conseguido; F-RPE: apertura de sutura media palatina fallida; CBCT: tomografia computarizada cone beam, M.B.: método de Bjork; A.F: análisis de Fishman; C.A.: clasificación de Angelieri; H.T: método de Hagg y Taranger; DP3: falange distal del tercer dedo; PP3: falange proximal del tercer dedo; MP3: falange mesial del tercer dedo, SMI: índice de maduración ósea; p: probabilidad; ϒs: coeficiente correlación de Spearman.

### Identificación de artículos

Se identificaron 365 artículos, de cuatro bases de datos: PubMed (94), Scopus (139), Embase (116) y ScienceDirect (16); posteriormente, se realizó una búsqueda manual y se encontró un artículo adicional. Se encontró y eliminó 88 artículos con Refworks y se excluyeron 209 artículos por título o resumen, como revisiones, reporte de casos, ensayos, estudios en animales, editoriales y comentarios. A la búsqueda de elegibilidad, se excluyeron 2 artículos en formato *no full-text* y 31 que no mostraban relación con el criterio de maduración de mano y muñeca, y la osificación de sutura media palatina. En la selección final se incluyó 4 artículos (Figura 1). 

### Características de diseños y demografía

Todos los estudios incluidos fueron aplicados a 3 poblaciones específicas: 1 en EE. UU. ^17^, 1 en Turquía ^20^ y 2 en Corea del Norte ^(21, 22)^. La distribución de diseños de estudio fue retrospectiva. Se excluyeron pacientes con enfermedades o ingesta de medicamentos que afectara al crecimiento óseo, así como pacientes con tratamiento de ortodoncia previo ^21^^,^^22^. El rango de la muestra osciló de 84 a 267 pacientes en total. El rango en las mujeres evaluadas fue de 45 a 135 y de los varones, de 39 a 132. El rango de edad de los pacientes evaluados osciló entre 7 y 18 años.

### Describir el estadio de maduración esquelética de la mano y muñeca según sexo y edad en adolescentes

Para la evaluación de maduración carpal con análisis de la mano y la muñeca se usaron el método de Bjork ^20^, el método de Hagg y Taranger ^22^ y el análisis de Fishman ^17^^,^^21^^,^^22^. Se observó predominio en cada etapa de maduración esquelética en el sexo femenino respecto del masculino, y que la maduración ósea se completó dos años antes en las mujeres que en los hombres.

### Caracterizar la osificación de la sutura media palatina según sexo y edad en adolescentes

Se evaluó la sutura media palatina mediante la observación de características en radiografía oclusal ^(17, 20)^ y tomografía *cone beam*^21^^,^^22^, mediante la clasificación de Angelieri ^14^^,^^17^^-^^22^. En la mayoría de los casos, el estadio A se presentó en edades menores de 10 años; el estadio B, en pacientes de 10 a 12 años; el estadio C, en pacientes que no pasaban de 14 años, y los estadios D y E, en pacientes mayores de 14 años. En los estadios iniciales no hubo diferencias según el sexo, pero desde el estadio C hubo mayor predominio en las mujeres en comparación con los hombres ^20^^-^^22^.

### Comparar el estadio de maduración esquelética de la mano y muñeca y la osificación de la sutura media palatina según sexo y edad en adolescentes

Según Revelo *et al*. ^17^, mientras aumenta progresivamente el grado de maduración ósea, se observa un aumento en la aproximación de la sutura media palatina en las radiografías oclusales, especialmente en etapas tardías de maduración, como ocurre en la etapa SMI9. Esto demuestra un aumento significativo cuando ocurre la aproximación de la sutura media palatina. Además, Jang *et al*. ^21^ hallaron que, entre el índice de maduración esquelética y la osificación de la sutura media palatina, existe una fuerte correlación (0,904), mediante la clasificación de análisis de Fishman y la clasificación de Angelieri, respectivamente, con mayor valor a diferencia de otros métodos de análisis. Esto indicaría que existe relación entre el aumento y progresión de la maduración ósea y la osificación de la sutura media palatina. 

En los resultados de análisis de tabulación cruzada entre la clasificación de Angelieri y el análisis de Fishman, con el tamaño de muestra y sexo, mostró un valor alto (γ = 0,924 > 0,905, t-b de Kendall = 0,087>0.784), con valores significativamente comparados con otros métodos de análisis. Además, Akbulut *et al*. (Turquía, 2020) señalan que existen cambios entre todos los elementos evaluados en un estudio carpal, antes y después del cierre de la sutura palatina, con correlación positiva en radio, MP3 y DP3 (p < 0,05), a excepción de PP3 (p < 0,063), y valores más significativos en radio (p < 0,001). Se encontró que los valores fractales son mayores cuando ocurre el cierre de la sutura palatina, por lo que se deduce que hay mayor mineralización en dicha zona. Yu et al. (22), en las etapas de maduración de la sutura media palatina, y la maduración esquelética de mano y muñeca, encontraron una correlación positiva (γ = 0,905; p < 0,05), así como una mayor relación de número de la población en SMI1 en estadio A, con un 84,4%, y en SMI11 en estadio E, con un 53,8% mediante la clasificación de Angelieri y el análisis de Fishman, correspondientemente. Además, se observó correlación positiva (γ = 0,830; p < 0,05), lo que indicaría ser confiable en las etapas de MP3, con el avance del cierre de sutura, mediante la clasificación de Angelieri y el método de Hagg y Taranger , respectivamente. Entre el análisis de Fishman y el método de Hagg y Taranger se encontró mayor valor de confiabilidad en los valores correspondientes en el primero.

## DISCUSIÓN

En los 4 artículos analizados, solo se realizaron los estudios en 3 poblaciones diferentes, a diferencia de otros estudios. El aumento del número de muestra llevaría a resultados más confiables para pasar a ser reproducibles ^17^^,^^20^^-^^22^. La evaluación en cada paciente dependerá de la experiencia y capacidad del especialista para determinar cuándo es el momento adecuado durante la etapa de crecimiento para iniciar el tratamiento de forma más eficiente ^1^^-^^5^^,^^8^^,^^23^. 

La práctica del análisis de la mano y muñeca es muchas veces ignorada, tal vez por la falta de familiarización con dicho análisis, por lo que suele ser desplazado con la evaluación de vértebras cervicales ^1^^-^^3^^,^^5^^,^^7^^,^^13^^,^^15^^,^^16^. Asimismo, este trabajo tuvo como objetivo considerar el análisis de la mano y muñeca como método *gold standard* para la confiabilidad en la predicción de la maduración de SMP ^1^^,^^13^^,^^16^^,^^22^. 

En unos estudios, se analizaron radiografías oclusales para evaluar el grado de separación de la SMP ^3^^-^^6^^,^^17^^,^^20^, a diferencia de otros que utilizaron una CBCT ^18^^-^^22^. En el segundo caso se encontró más información sobre las características morfológicas propias de cada estadio de osificación. Asimismo, Anghelieri *et al*. sugieren que la maduración de la sutura media palatina se puede clasificar en cinco etapas (etapas A-E). Mediante la observación de imágenes CBCT, para la evaluación de la sutura media palatina, descubrieron que era posible minimizar el fracaso de la ampliación del maxilar en los casos de pacientes adolescentes y adultos jóvenes ^3^^,^^4^^,^^6^^,^^14^^,^^17^^,^^22^^,^^24^^-^^26^. 

En un estudio se compararon tres tipos análisis de medición de mano y muñeca ^21^, y el análisis de Fishman fue el que tuvo mayor resultado de confiabilidad, a diferencia del método de Björk y el de Hagg y Taranger, que no son comúnmente usados. Fishman *et al*. desarrollaron una modalidad para evaluar las etapas de maduración esquelética en base a 11 indicadores de madurez esquelética (SMI) ^7^^,^^10^^-^^17^^,^^20^^-^^22^. Se considera desde SMI 1-4 el momento ideal para realizar cambios ortopédicos y evitar efectos que causen algún daño, ya que estos se encuentran aún sin evidencia de fusión en la SMP, característicos propios de los estadios A-B ^17^^,^^20^^-^^22^.

Se encontró que, mientras hay un aumento en el desarrollo esquelético, como sucede a partir de SMI7, además del incremento en la aproximación y fusión de la sutura media palatina, estos fueron compatibles desde el inicio del estadio C. Así como en otros estudios, se evaluaron las características en relación con la maduración esquelética, la sutura, el sexo y la edad. Y solo en un estudio se realizó la evaluación con la separación de la sutura, lo que limitaría la validez de los resultados a diferencia de los demás. Ahora es posible observar imágenes de la sutura del paladar mediante una tomografía computarizada de haz cónico (CBCT), lo que resultaría difícil de observar en las radiografías convencionales ^24^^-^^26^. Sería idóneo que desde SMI7-9 se utilice una CBCT para tomar la decisión idónea, ya que se encontraría en estadio C-D, lo que sería útil en los casos que se necesitase tomar una decisión para el plan de tratamiento.

Mientras el tratamiento sea interceptivo, es más eficaz durante la infancia porque las suturas aún no se encuentran fusionadas ^17^^-^^22^^,^^24^^-^^26^. Por otro lado, en los SMI10-11 ya alcanzaría el desarrollo madurativo esquelético siendo compatibles con el estadio D-E de la sutura media palatina. Dichas etapas serían compatibles con procedimientos que requieran una expansión quirúrgica asistida.

Con base a la detallada revisión de literatura que se realizó, nuestro estudio ha demostrado que existen pocas investigaciones relacionadas con el tema, además de la escasa población estudiada. Esto se podría considerar para la elaboración de futuros trabajos de investigación de corte clínico.

## CONCLUSIONES

A partir de en los resultados de esta revisión de la literatura, con respecto a la relación del estadio de maduración de mano y muñeca, y la morfología de la SMP y viceversa en adolescentes, se demostró que tienen correlaciones altas y positivas, lo que llevaría a concluir que los métodos de evaluación para el diagnóstico mediante el análisis carpal pueden usarse para las evaluaciones de predicción del estadio de maduración de la SMP.

## Fuente de financiamiento:

Autofinanciado
